# Assessment of the Protective Effect of* Lepidium sativum* against Aluminum-Induced Liver and Kidney Effects in Albino Rat

**DOI:** 10.1155/2019/4516730

**Published:** 2019-07-18

**Authors:** Maha Jameal Balgoon

**Affiliations:** Department of Biochemistry, Faculty of Science, King Abdulaziz University, Saudi Arabia

## Abstract

**Background and Objectives:**

Environmental pollution with the different Aluminum (Al) containing compounds has been increased. Liver and kidney are two vital organs targeted by Al accumulation. The aim of this study was to assess the possible protective and curative effects of* Lepidium sativum *Linn (LS) against Al-induced impairment of liver and kidney in albino rat and to explore the mechanism behind this effect.

**Materials and Methods:**

This experimental animal-based study included fifty albino rats divided into five groups, the control, LS-treated (20 mg/kg), AlCl_3_-treated (10 mg/kg), AlCl_3_ then LS, and AlCl_3_ plus LS-treated, simultaneously for 8 weeks. At the end of the experiment, hepatic and renal functions as well as the biomarkers of antioxidants activities were assessed in the serum. Both liver and kidney were dissected out and histopathologically examined.

**Results:**

This study showed that administration of AlCl_3_ caused a significant (p<0.05) reduction in rats body weight. It significantly increased serum AST, ALT, ALP, bilirubin, urea, and creatinine levels and decreased total protein and albumin. AlCl_3_ significantly reduced enzymatic (catalase), nonenzymatic (reduced glutathione), and ferric reducing antioxidant power (FRAP) in the serum. Histopathologically, it induced necrosis and degeneration of hepatocytes, glomeruli, and renal tubules. Administration of LS after or along with AlCl_3_ significantly restored the serum biomarkers of liver and kidney functions to their near-normal levels and had the ability to overcome Al-induced oxidative stress and preserved, to some extent, the normal hepatic and renal structure. The coadministration of LS had a superior effect in alleviating Al-induced changes.

**Conclusion:**

Exposure to AlCl_3_ induced a set of functional and structural changes in the liver and kidney of rats evident through both biochemical and histopathological assessment. The antioxidant activity of LS seeds mediated a protective and curative effect of LS against such changes. Further study through a rigorous clinical trial to prove LS activity on human is recommended.

## 1. Introduction

Aluminum (Al) is among the most abundant elements on the earth. Absorption or accumulation of Al in humans occurs via diet as in some food products and additives medication like antacids vaccines and parenteral fluids, adding to cosmetics, inhaled fumes, and particles from occupational exposures [1]. It was believed that Al was nontoxic and was quickly excreted in the urine so it was widely used in daily life. Though, it was known later on that it negatively affects human health [2]. The Agency for Toxic Substances and Disease Registry (ATSDR) reported that Aluminum is mainly distributed in the bone, liver, testis, kidneys, and brain [3]. This metal disrupts the prooxidant/antioxidant balance in tissues leading to biochemical and physiological dysfunctions due to an excessive reactive oxygen species (ROS) generation [4].

*Lepidium sativum *Linn (LS) is an edible annual herb which belongs to the family Brassicaceae, cultivated throughout India, Europe, and the United States as well as Arabian countries. It has many names like Asaliyo, pepper cress, and Elrshad [5]. The seeds and leaves of the plant have volatile oils and are a good source of amino acids, minerals, and fatty acids. They have the ability to act in vitro as antioxidants due to their high content of phenolic compounds, and thus they could have potential preventive effects towards chronic diseases [6]. LS seeds are used in Saudi folk medicine for multiple applications, but mainly in fracture healing and after maternity [7]. 

The methanolic extract of LS seeds is described to have hepatoprotective activity in rats as it significantly reduced the elevated liver enzymes and improved the severe fatty changes in the liver induced by carbon tetrachloride (CCl_4_) [8]. In another study ethanolic extracts of LS seeds were proved to have nephrocurative and nephroprotective activity as it reduced urea and creatinine level in serum of cisplatin-induced model of nephrotoxicity [9]. In previous toxicological studies, safety of LS seeds was reported [10]. So LS was selected in this study to investigate its ability to protect against Aluminum-induced kidney and hepatotoxicity. Therefore, this study was carried out to evaluate the protective and therapeutic potential of LS against the hepatic and renal functional and structural changes caused by AlCl_3_ and to explore the mechanism behind these possible effects.

## 2. Materials and Methods

### 2.1. Chemicals

All chemicals, used in this study, were of analytical grade. Aluminum chloride (AlCl_3_) was purchased from Sigma Chemical Company, St. Louis, Missouri, USA. Seeds of* Lepidium sativum* (Family: Cruciferae) were obtained from the local herbalist shops in Jeddah. These seeds were previously characterized in a study conducted by Raouf et al. in the King Fahd Medical Research Center (KFMRC), King Abdulaziz University, Jeddah, Kingdom of Saudi Arabia [11], and their aqueous extracts were prepared according to the method described by Eddouks et al. [12].

### 2.2. Animals

An ethical approval of this experimental study was obtained from the biomedical research ethics committee at the Faculty of Medicine, King Abdulaziz University, Jeddah, Saudi Arabia.

Fifty albino rats with average body weight from 200 to 250 g were utilized in this study. They were obtained from the KFMRC and were left to acclimatize before the experiment for one week; then they were randomly divided into five groups (n=10 each).

The first group included the control rats which were intraperitoneally (i.p.) injected of 1 ml of normal saline and given 1 ml of normal saline by oral gavage for 8 weeks.

Rats of the second group (LS-treated group) were given water extract of LS daily at a dose 20 mg/kg in 1 ml of normal saline by oral gavage in addition to i.p. injection of normal saline.

Rats of the third group (AlCl_3_-treated group) were i.p. injected with AlCl_3_ at a dose of 10 mg/kg of body weight dissolved in 1 ml of distilled water daily for 8 weeks [13].

Rats of the fourth group (AlCl_3_ then LS group) were i.p. injected with AlCl_3_ at the same dose for 8 weeks and then given water extract of LS by oral gavage at the same dose daily for another 4 weeks [14].

Rats of the fifth group (AlCl_3_ plus LS group) were i.p. injected with AlCl_3_ simultaneously with giving water extract of LS by oral gavage at the same dose for 8 weeks.

At the end of the experiment, rats were fasted overnight. The body weight of the rats was measured. Then, rats were sacrificed by cervical decapitation, under light anesthesia with diethyl ether, and the blood samples were collected in plain tubes, centrifuged at 4000 rpm for 15 minutes to obtain serum, and stored at - 20°C till analysis. Livers and kidneys were rapidly dissected out and processed for histopathological examination.

### 2.3. Biochemical Assessment

Alanine aminotransferase (ALT) and aspartate aminotransferase (AST) were colorimetrically assessed according to the method of Reitman and Frankel [15] while serum alkaline phosphatase (ALP) was measured according to the method described by Belfield and Goldberg [16]. Total bilirubin, proteins, and albumin were measured according to the methods of Walter and Gerade [17] and Lowry et al. [18], respectively. Total cholesterol, triglycerides, and low-density lipoprotein cholesterol (LDLc) were determined as previously described by Richmond and Fossati and Principle [19, 20]. Reduced glutathione (GSH) level and enzymatic antioxidant catalase (CAT) activity were also assessed as previously described [21]. Ferric Reducing Antioxidant Power (FRAP), an antioxidant capacity indicator, was assessed using FRAP assay Kit (Cell Biolabs, USA, Cat. no. STA-859). Creatinine and urea in serum were measured using Chronolab kits (Spain).

### 2.4. Histopathological Examination

Parts of livers and kidneys of all rats were fixed in 10% buffered neutral formalin solution and further processed to obtain of paraffin blocks. Five-micron thick paraffin sections were prepared and routinely stained with Hematoxylin and Eosin (H&E) and examined for the histopathological changes using the light microscope [22].

### 2.5. Statistical Analysis

The data obtained were statistically analyzed by using the Statistical Package of Social Science (SPSS) version 20 (SPSS Inc., Chicago, IL). The results were expressed as mean ± standard deviation (SD). One Way ANOVA followed by (LSD) post hoc test was used to assess the statistical significance between the different groups. A* p*<0.05 was accepted as statistically significant.

## 3. Results

It was noticed that final body weights measured at the end of the experiment as well as the percentage of change in body weight were significantly lower in both groups receiving AlCl_3_ alone or along with LS compared to the control. Both parameters were significantly higher in all groups receiving LS, compared to those receiving AlCl_3_ alone. The final body weight and the percentage of change in body weight were significantly lower (P=0.001; p=0.03) in rats receiving AlCl_3_ plus LS compared to those receiving AlCl_3_ then LS, respectively (Table 1).

### 3.1. Biochemical Results

Serum cholesterol, triglyceride, and LDL-C levels were significantly lower in the groups receiving LS alone or with AlCl_3_ compared with the group receiving AlCl_3_ alone. There was no significant difference in these parameters in rats receiving AlCl_3_ plus LS compared to those receiving AlCl_3_ then LS (Table 2).

FRAP and reduced glutathione levels were significantly increased (p=0.001) in rats receiving LS compared to the control. On the other hand, these two parameters as well as catalase level were significantly reduced in AlCl_3_-treated group. FRAP and catalase levels were significantly increased in rats receiving AlCl_3_ then LS and AlCl_3_ plus LS compared to those receiving AlCl_3_ alone. Receiving LS along with AlCl_3_ has a more significant effect on increasing catalase and reduced glutathione levels (Table 3).

Serum AST, ALT, and ALP levels were significantly higher (p=0.001) in rats receiving AlCl_3_ compared to the control. These levels were significantly reduced in groups receiving AlCl_3_ then LS as well as AlCl_3_ plus LS compared with AlCl_3_ group with no significant difference between the two groups (Table 4).

Total proteins and albumin levels were significantly reduced in rats receiving AlCl_3_ compared to the control and they were significantly increased in groups receiving AlCl_3_ then LS as well as AlCl_3_ plus LS compared with AlCl_3_ group with no significant difference between the two groups (Table 4).

Serum total bilirubin, creatinine, and urea levels were significantly higher (p=0.001) in rats receiving AlCl_3_ while they significantly decreased in groups receiving AlCl_3_ then LS as well as AlCl3 plus LS compared with AlCl_3_ group (Tables 4 and 5).

### 3.2. Histopathological Results

**Figure 1:**
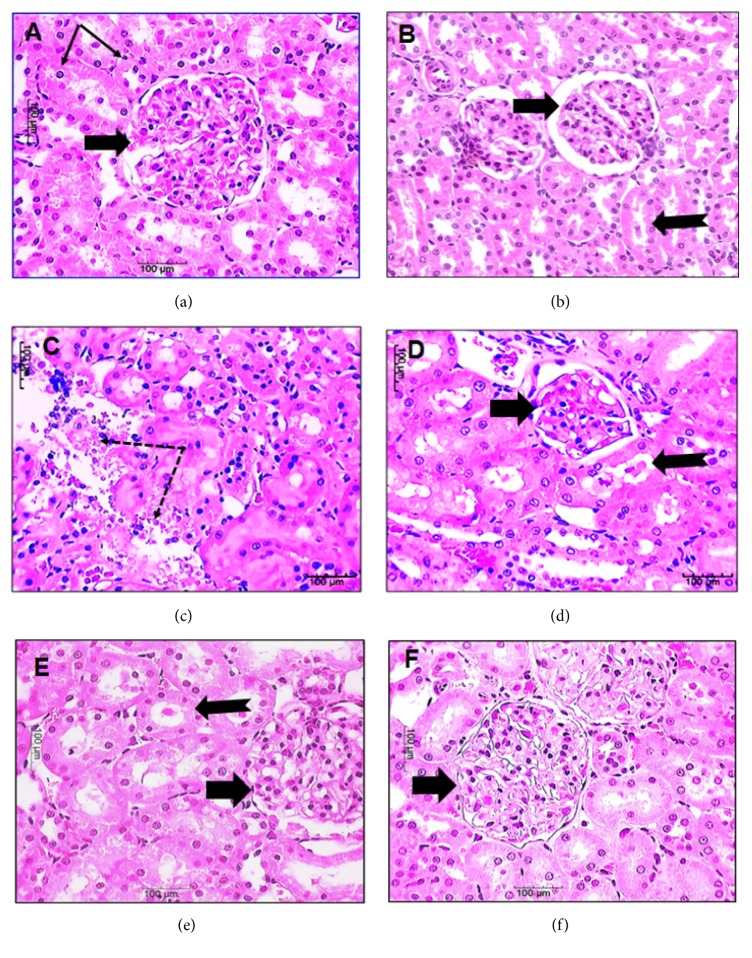
Sections of kidney of control rats (a) and rats receiving LS show intact renal cortex with intact renal corpuscle (arrows) and renal tubules. Renal cortex of rats receiving AlCl_3_ (b-d) showing shrunken renal corpuscles (arrows) with marked hypocellularity and atrophied glomeruli. Renal tubules showed dilated lumina with hyaline casts (bifid arrows) and in some of them appear completely distorted (dotted arrows). Renal cortex of rats receiving AlCl_3_ then LS (e) showing intact renal corpuscles with preserved cellularity (arrow) and intact renal tubules apart from few with dilated lumina and hyaline casts (thin arrows). Renal cortex of rats receiving AlCl_3_ plus LS (f) showing intact structure of renal cortex (H&E X 400, Bar=100). LS:* Lepidium sativum*.

It was observed that, in this study, kidney of control rats and those receiving LS had intact structure. On the other hand, renal cortex of rats receiving AlCl_3_ showed shrunken renal corpuscles with marked hypocellularity and atrophied glomeruli. The renal tubules appeared dilated with hyaline casts in their lumina and some of them appear completely distorted. Rats receiving AlCl_3_ then LS showed intact renal cortex, preserved cellularity of renal corpuscles, and intact renal tubules apart from few with dilated lumina and hyaline casts. Renal cortex of rats receiving AlCl_3_ plus LS had intact renal cortex (Figure 1).

**Figure 2:**
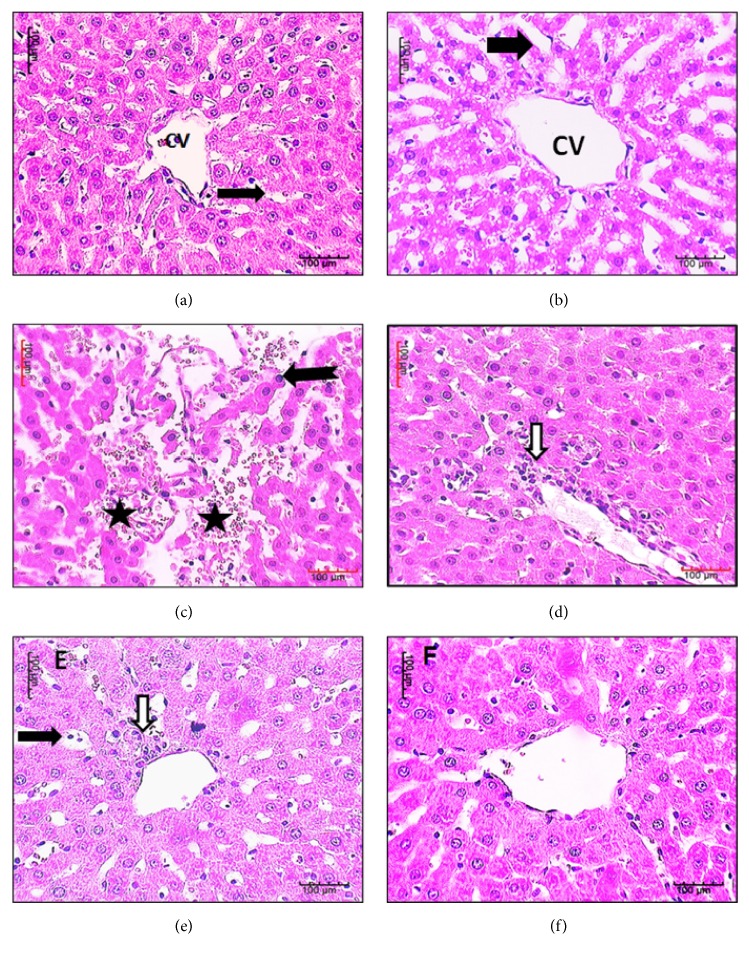
Sections in liver of control (a) and rats receiving LS (b) show intact liver apart from some dilated blood sinusoids (arrow). Liver of rats receiving AlCl_3_ (c, d) showing massive focal hepatic necrosis with shrunken hepatocytes (bifid arrows). Damaged sinusoids with extravasation of blood cells (stars) and perivascular cell necrosis and inflammatory cells (white arrow). Liver of rats receiving AlCl_3_ then LS (e) showing intact structure apart from some dilated sinusoids (arrows) associated with few inflammatory cells (white arrow) while livers of rat receiving AlCl_3_ plus LS (f) appear intact (H&E X 400, Bar=100). LS:* Lepidium sativum*.

When it came to the liver, it was observed that both control rats and those receiving LS showed intact liver. On the other hand, liver of rats receiving AlCl_3_ showed massive focal hepatic necrosis with shrunken hepatocytes, damaged sinusoids with extravasation of blood cells, perivascular cell necrosis, and inflammatory cells. Liver of rats receiving AlCl_3_ then LS showed intact structure apart from some dilated sinusoids associated with few inflammatory cells while liver of rats receiving AlCl_3_ plus LS appeared intact (Figure 2).

## 4. Discussion

Aluminum chloride was considered a nontoxic metal for a long time, but more attention has been focused on its adverse effects on human and animal health [23]. Oxidative stress is considered to be a major contributor, a trigger for sever metal toxicities, and has been reported to be associated with long retention of metals in some tissues [24]. It was well known that an imbalance between the overproduction of reactive oxygen species (ROS) and elimination of free radicals induces oxidative stress [4]. This study assessed the impact of AlCl_3_ on the function and structure of the liver and kidney, the possible protective and therapeutic effects of LS against this impact, and the mechanism behind these effects.

AlCl_3_ was frequently reported to reduce body weight when administrated in rats [25]. This is evident also in this study. LS administration during or after AlCl_3_ was found to protect against weight reduction and changes in weight percentage induced by AlCl_3_ in this study. In a previous study, Sahane et al. observed that LS prevented weight loss in diabetic rats [26].

In the present study, the significant disturbing effect of AlCl_3_ administration on the lipid profile was evident as this was in agreement with previous studies of Ghorbel et al. who reported that AlCl_3_ induced abnormal activities of lipase enzymes that seem to be one of the chief factors responsible for the cholesterol rise in serum after AlCl_3_ administration [4]. In an earlier study, Al-Hashem reported that the increased cholesterol and triglycerides induced by AlCl_3_ indicated lost membrane integrity, disturbance of lipid metabolism, and/or liver dysfunction [27]. When it came to the effect of LS on the lipid profile, it was observed in this study that LS succeeded to reduce AlCl_3_-induced elevation in the lipid profile. This was previously reported by Al-Khazraji on a diabetic animal model [28]. This hypolipidemic effect of LS might be attributed to its possible inhibitory effect on the endogenous synthesis of lipids. In a more recent study, both hypoglycemic and hypolipidemic effects of LS seeds extract administrated at a dose of 20 mg/k were reported by Kamani et al. and they attributed these effects to the antioxidant activity of LS [29].

In the present study, assessment of antioxidants, mainly reduced glutathione (GSH), catalase, and FRAP, showed that AlCl_3_ treatment resulted in significant decrease in these parameters. This decrease was significantly alleviated by administration of LS seed aqueous extract. Significant differences in catalase and GSH levels in rats receiving LS along with AlCl_3_ compared to those receiving LS after AlCl_3_ indicated a more potent protective effect of LS. It was said that accumulation of Aluminum in organism can increase lipid peroxidation rates [30]. Lipid peroxidation is a chemical mechanism capable of disrupting the structure and the function of the biological membranes that occurs as a result of free radical attack on lipids [31]. In the present study, it was observed that LS repaired the oxidant/antioxidant balance as it significantly increased the antioxidants GSH, CAT, and FRAP in the serum. It was reported that LS and its metabolites are powerful antioxidants that can directly detoxify free radicals species by electron donation. It can also defend indirectly against oxidative damage by repair of the antioxidant system through enhancing the activities of a variety of antioxidants like GSH, CAT, and FRAP [32].

Exposure to AlCl_3_, in the present study, resulted in alterations in the liver and kidney function and structure evident through both biochemical and histopathological assessment, respectively. Hepatic necrosis and degenerated and inflammatory changes were noted in livers of rats receiving AlCl_3_. The exposure to AlCl_3_ significantly increased serum ALT, AST, ALP, and bilirubin and decreased total protein especially the albumin when compared to the control. This was in agreement with the findings of Imam et al. [23]. Liver manages a variety of metabolic substances and hepatocytes are easily affected by a variety of factors and harmful products like exposure to high doses of Aluminum, which proved to be accumulated in the liver and cause alterations of the hepatic function. Degeneration, inflammation, and necrosis caused by hepatocyte damage can lead to an increase in the permeability of cell membranes with subsequent increase of AST and ALT into the blood, which are known to be effective indicators of liver damage [33].

Imam et al. reported that increased serum total bilirubin in the of AlCl_3_-treated rats may be the result of decreased liver uptake (conjugation), increased production from hemolysis, and increased free radical production or due to the onset of periportal necrosis as proved histopathologically [23]. They added that decreased albumin may be due to changes in protein synthesis and/or metabolism in the liver. Furthermore, Al may promote proteinuria by causing a nephritic syndrome or chronic glomerulonephritis [27].

The role of antioxidant activity of LS seed aqueous extract against AlCl_3_–induced alternations in both liver and kidney was also assessed. The presence of flavanoids, triterpens, alkaloid, tannin, and coumarins in LS explains its role in hepatoprotection by inhibiting the toxic radicals mediated damage. Our result agrees or corresponds with previous published results or studies [23].

Kidney is among the tissues in which Al accumulates. This accumulation promotes degeneration in the renal tubular cells and resulted in nephrotoxicity [34]. In this study, creatinine and urea were assessed as they are significant indicators of renal function. AlCl_3_ increased both parameters indicating impaired renal function as a result of glomeruli and tubules are damaged, which was evident histopathologically. This was in accordance with the findings of Joshi et al. and Imam et al. [23, 35]. Based on previous recommendations, LS was advised for treatment of hypertension, diabetes, and renal disease [5]. In this study, the protective and curative effect of ethanolic extract of LS on the renal function was observed as it significantly reduced the level of urea and creatinine, indicating increased glomerular filtration rate. Al Hamedan reported a diuretic activity of LS extract when administrated daily for 3 weeks [36].

In conclusion, exposure to AlCl_3_, at a dose of 10 mg/kg of body weight daily for 8 weeks, induced a set of functional and structural changes in the liver and kidney of rats evident through both biochemical and histopathological assessment, respectively. The antioxidant activity of LS seed aqueous extract, at a dose of 20 mg/kg for 8 weeks, mediated a protective and curative effect of LS against AlCl_3_-induced hepatic and renal changes. Further study, through a rigorous clinical trial to prove such effect of LS on human, is recommended.

## Figures and Tables

**Table 1:** Comparison of body weights in different studied groups.

Parameters	Control	LS-treated	AlCl_3_-treated	AlCl_3_ then LS	AlCl_3_ plus LS
Initial body weight (g)	224.78±17.33	212.56±14.78	219.67±17.1	228.56±26.8	212.56±14.78

		P#=0.173	P#=0.565	P#=0.670 P##=0.319	P#=0.173 P##=0.425 P###=0.077

Final body weight (g)	316.78±13.45	326.11±29.15	227.56±17.5	309.44±40.1	251.56±10.7

Significance		P#=0.429	P#<0.001	P#=0.533 P##<0.001	P#=0.001 P##=0.046 P###<0.001

Weight gain (g)	92.00±18.61	110.22±33.41	7.67±2.87	69.78±29.6	39.00±14.07

Significance		P#=0.094	P#<0.001	P#=0.043 P##<0.001	P#<0.001 P##=0.005 P###=0.006

Percentage changes in body weight (%)	41.56±10.88	52.29±17.46	3.51±1.40	30.69±13.23	18.72±7.81

Significance		P#=0.055	P#<0.001	P#=0.052 P##<0.001	P#<0.001 P##=0.008 P###=0.033

Data are expressed as mean±standard deviation. Comparison was made using One Way ANOVA test (LSD). P#: p value of comparison versus control; P##: p value of comparison versus AlCl_3_ group; P###: p value of comparison versus AlCl_3_ then LS group.

**Table 2:** Comparison of lipid profiles in different studied groups.

Parameters	Control	LS-treated	AlCl_3_-treated	AlCl_3_ then LS	AlCl_3_ plus LS
Cholesterol (mg/100ml)	80.51±9.52	73.24±3.99	106.03±12.75	88.33±3.92	86.24±6.74

Significance		P#=0.104	P#<0.001	P#=0.082 P##<0.001	P#=0.056 P##=0.019 P###=0.184

Triglyceride (mg/100ml)	66.84±8.47	58.01±13.22	99.21±21.21	71.37±10.34	81.24±10.86

Significance		P#=0.233	P#<0.001	P#=0.537 P##=0.001	P#=0.197 P##<0.001 P###=0.634

LDL-C (mg/100ml)	17.17±3.02	14.91±1.46	21.04±2.90	19.03±1.46	18.19±1.28

Significance		P#=0.060	P#=0.002	P#=0.119 P##=0.092	P#=0.388 P##=0.020 P###=0.472

Data are expressed as mean +/- standard deviation. Comparison was made using One Way ANOVA test (LSD). P#: p value of comparison versus control; P##: p value of comparison versus AlCl_3_ group; P###: p value of comparison versus AlCl_3_ then LS group.

**Table 3:** Comparison of oxidative stress markers in the serum of different studied groups.

Parameters	Control	LS-treated	AlCl_3_-treated	AlCl_3_ then LS	AlCl_3_ plus LS
FRAP (*μ*U/L)	82.20±13.33	150.80±15.99	45.40±5.68	65.20±9.88	67.80±7.79

Significance		P#<0.001	P#<0.001	P#=0.026 P##=0.01	P#=0.055 P##=0.005 P###=0.717

Catalase (*μ*U/L)	97.60±6.11	99.80±3.11	28.60±9.45	57.40±10.74	83.00±14.78

Significance		P#=0.724	P#<0.001	P#<0.001 P##<0.001	P#=0.027 P##<0.001 P###<0.001

Reduced glutathione (nmol/L)	2.81±1.09	6.47±1.51	0.55±0.25	1.00±0.27	2.29±0.79

Significance		P#<0.001	P#<0.001	P#=0.005 P##=0.457	P#=0.379 P##=0.008 P###=0.039

Data are expressed as mean +/- standard deviation. Comparison was made using One Way ANOVA test (LSD). P#: p value of comparison versus control; P##: p value of comparison versus AlCl_3_ group; P###: p value of comparison versus AlCl_3_ then LS group.

**Table 4:** Comparison of liver functions in the serum of different studied groups.

Parameters	Control	LS-treated	AlCl_3_-treated	AlCl_3_ then LS	AlCl_3_ plus LS
AST (U/L)	105.82±11.02	104.36±25.41	154.43±22.26	131.63±26.15	133.25±7.12

Significance		P#=0.871	P#=0.001	P#<0.006 P##=0.014	P#=0.004 P##=0.022 P###=0.857

ALT (U/L)	61.41±5.50	55.17±7.79	101.12±6.64	78.93±9.60	84.19±5.91

Significance		P#=0.060	P#<0.001	P#<0.001 P##<0.001	P#<0.001 P##<0.001 P###=0.111

ALP (U/L)	193.00±22.98	183.10±11.97	92.50±14.21	140.70±15.84	138.00±19.11

Significance		P#=0.206	P#<0.001	P#<0.001 P##<0.001	P#<0.001 P##<0.001 P###=0.728

Total proteins (g/L)	5.90±0.31	5.80±0.30	5.31±0.18	5.62±0.32	5.68±0.19

Significance		P#=0.41	P#<0.001	P#=0.03 P##=0.013	P#=0.072 P##=0.004 P###=0.642

Albumin (g/L)	3.10±0.26	3.12±0.22	2.80±0.12	3.02±0.15	2.96±0.13

Significance		P#=0.798	P#<0.001	P#=0.332 P##=0.008	P#=0.082 P##=0.055 P###=0.430

Total bilirubin (U/L)	0.057±0.009	0.049±0.007	0.090±0.015	0.064±0.022	0.073±0.013

Significance		P#=0.25	P#<0.001	P#=0.29 P##<0.001	P#=0.015 P##=0.012 P###=0.156

Data are expressed as mean +/- standard deviation. Comparison was made using One Way ANOVA test (LSD). P#: p value of comparison versus control; P##: p value of comparison versus AlCl_3_ group; P###: p value of comparison versus AlCl_3_ then LS group.

**Table 5:** Comparison of kidney function tests in the serum of different studied groups.

Parameters	Control	LS-treated	AlCl_3_-treated	AlCl_3_ then LS	AlCl_3_ plus LS
Creatinine (mmol/L)	0.30±0.02	0.29±0.04	0.45±0.11	0.31±0.02	0.33±0.05

Significance		P#=0.869	P#<0.001	P#=0.681 P##<0.001	P#=0.206 P##<0.001 P###=0.389

Urea (mmol/L)	36.21±2.57	36.00±4.62	52.23±4.76	44.06±6.43	48.46±8.17

Significance		P#=0.944	P#<0.001	P#=0.014 P##=0.011	P#<0.001 P##=0.220 P###=0.154

Data are expressed as mean +/- standard deviation. Comparison was made using One Way ANOVA test (LSD). P#: p value of comparison versus control; P##: p value of comparison versus AlCl_3_ group; P###: p value of comparison versus AlCl_3_ then LS group.

## Data Availability

The data used to support the findings of this study are included within the article.
